# *bz-rates*: A Web Tool to Estimate Mutation Rates from Fluctuation Analysis

**DOI:** 10.1534/g3.115.019836

**Published:** 2015-09-02

**Authors:** Alexandre Gillet-Markowska, Guillaume Louvel, Gilles Fischer

**Affiliations:** Sorbonne Universités, UPMC Univ. Paris 06, Institut de Biologie Paris-Seine UMR7238, Biologie Computationnelle et Quantitative, F-75005, Paris, France

**Keywords:** mutation rate, fluctuation assay, Luria-Delbrück

## Abstract

Fluctuation analysis is the standard experimental method for measuring mutation rates in micro-organisms. The appearance of mutants is classically described by a Luria-Delbrück distribution composed of two parameters: the number of mutations per culture (*m*) and the differential growth rate between mutant and wild-type cells (*b*). A precise estimation of these two parameters is a prerequisite to the calculation of the mutation rate. Here, we developed *bz-rates*, a Web tool to calculate mutation rates that provides three useful advances over existing Web tools. First, it allows taking into account *b*, the differential growth rate between mutant and wild-type cells, in the estimation of *m* with the generating function. Second, *bz-rates* allows the user to take into account a deviation from the Luria-Delbrück distribution called *z*, the plating efficiency, in the estimation of *m*. Finally, the Web site provides a graphical visualization of the goodness-of-fit between the experimental data and the model. *bz-rates* is accessible at http://www.lcqb.upmc.fr/bzrates.

A classical approach to calculate mutation rates (*μ*) in micro-organisms consists in performing fluctuation analyses through multiple cultures grown in parallel under identical conditions (Luria and Delbrück 1943). Each individual culture is started with a small inoculum such that the mutational events that occur during the culture are independent. Cultures are then plated on selective media to determine the number of mutants present in each culture. Estimating the mutation rate from these experimental data is of great interest for biologists and has been the object of many mathematical developments [for review, see Foster (2006)].

Calculating mutation rates requires to first estimate the mean number of mutations per culture (*m*) under the assumptions of a Luria-Delbrück distribution model (Lea and Coulson 1949). Once a value of *m* has been calculated, the mutation rate μ can be easily inferred by dividing *m* by the total number of cells in the culture [although this can lead to an underestimation of the mutation rate (Ycart and Veziris 2014)]. Most of the available estimators rely on the maximum likelihood (ML), method which was shown to be accurate for recovering *m* values (Sarkar *et al.* 1992; Jones 1994; Stewart 1994; Jaeger and Sarkar 1995; Zheng 2002; Gerrish 2008). However, ML estimators can become unstable for fluctuation assays involving cultures with large numbers of mutants. In such cases, the empirical probability generating function (GF) remains robust and is preferred over ML (Hamon and Ycart 2012).

One major parameter affecting the estimation of *m* is *b*, the differential fitness between mutant and wild type cells (*i.e.*, the ratio between the mutant and the wild-type growth rates). In the case of differential growth rate, several estimators that jointly calculate *m* and *b* have long been made available (Koch 1982; Jones 1994; Jaeger and Sarkar 1995; Zheng 2002, 2005; Hamon and Ycart 2012). The code of these estimators is easily accessible but requires running command lines or installing third-party tools.

In addition, the estimation of *m* also can be affected by another parameter: the plating efficiency, *z*. This criterion is defined as the fraction of the cultures that is plated on selective media. This parameter accounts for the fact that not all mutants are experimentally detected when only a fraction of the cultures is plated.

Here we propose a new integrated Web tool called *bz-rates*, which provides three useful advances over the only Web tool available to estimate *m* (Hall *et al.* 2009). First, it allows taking into account *b*, the differential growth rate between mutant and wild-type cells, in the estimation of *m* with the GF. Note that *bz-rates* does not propose new mathematical developments but fully relies on the available GF estimator. Second, *bz-rates* allows the user to take into account the *z* deviation in the estimation of *m* by using the formulation suggested in (Foster 2006) and initially proposed by Stewart *et al.* (1990). Note that more recent formal mathematical treatments to this problem also are available but were not implemented here (Stewart 1991; Jones 1993; Zheng 2008a). Finally, *bz-rates* computes the goodness-of-fit [as described in Boe *et al.* (1994)] between the experimental data and the two-parameter Luria-Delbrück model and provides the user with a graphical visualization of the fit.

## Materials and Methods

### Fluctuation assay

Fluctuation assays were performed with a BY4741 yeast strain (*TRP1Δ*5′(1-362)::*natNT2*, *CYC1Δ*::*TRP1Δ*3′(864-958)-*hph*, *ura3*, *clb5Δ*::*KanMX4*) carrying two nonfunctional alleles of the *TRP1* gene involved in tryptophan biosynthesis on two different chromosomes. One copy is truncated in 3′ and the other copy in 5′ leaving a 400-bp homology region repeated in the two alleles. A nonallelic homologous recombination event between the two heteroalleles generates a reciprocal translocation that restores tryptophan prototrophy. These mutant cells can therefore be easily selected by plating the cultures onto standard complete synthetic media depleted for tryptophan. To summarize in brief, 30 parallel cultures (500 μL) were started by inoculating into rich media (Broth Yeast Extract-Peptone-Dextrose) ∼100 cells per well in a 2-mL deep-well plate. Cells were grown without agitation at 30° until they reached an optical density of 0.85 (6 × 10^6^ cells/mL) and plates were incubated for 4 d at 30° before counting the number of mutants per plate.

### Growth rates

The growth rate of three independent mutants and wild-type cells was measured by growth curve experiments in 100 μL of rich media with a Tecan Sunrise robot in triplicates.

### Data availability

*bz-rates* was developed in Python with the Django v1.6 framework. It is a free Web tool distributed under the terms of the GNU General Public License. *bz-rates* is accessible at http://www.lcqb.upmc.fr/bzrates. The source code, available at https://github.com/gillet/bzrates, can be easily modified to implement other estimators and clones of the tool can be set up elsewhere.

## Results

### Implementation

*bz-rates* uses the empirical probability GF estimator from Hamon and Ycart (2012) and Ycart (2013). This method allows a precise estimation of *m* across a larger range of parameter values than the ML method. The cellular division time model chosen in *bz-rates* is not the classical exponential model but a constant division time model (Dirac). Although there is no universal cellular division time model because it depends on experimental conditions like the strain or the media, the Dirac model usually is the most accurate for the estimation of *b* and as accurate as the exponential model for the estimation of *m*. Note that this division time model induces a positive bias in the estimation of large values of *m* (Ycart 2013).

When *b* is known, the value provided by the user is used to estimate *m* with the GF. However, when the mutant relative fitness *b* is not known, *bz-rates* estimates both *m* and *b* with the GF function.

The *m_corr_* value that takes into consideration the plating efficiency *z* is calculated according to equation (41) in (Stewart *et al.* 1990): $m_{corr}=m\cdot \left(z-1\right)/\left(z\cdot ln\left(z\right)\right)$. μ is defined as $m/\bar{Np}$ and μ_*corr*_ as $m_{corr}/\bar{Nt}$, where $\bar{Np}$ and $\bar{Nt}$ represent the mean number of cells per plate and per culture, respectively. $CL_{lower}$ and $CL_{upper}$ provide the lower and upper confidence limits of *m_corr_* (level of confidence = 95%). σ*_Nc_* provides the standard deviation of the number plated cells.

To test the goodness-of-fit of the data to the model, *bz-rates* performs a Pearson’s chi-square test. The value of χ^*2*^ gives the Pearson’s chi-square goodness-of-fit and the χ^*2*^ − *pval* its associated p-value. The null hypothesis is rejected in the case χ^*2*^ − *pval* < 0.01 meaning that the cumulative distribution function does not fit with the experimental data (empirical cumulative distribution function). In this case, the user is warned that the estimation of the mutation rate is not reliable.

### Interface

**Figure 1:**
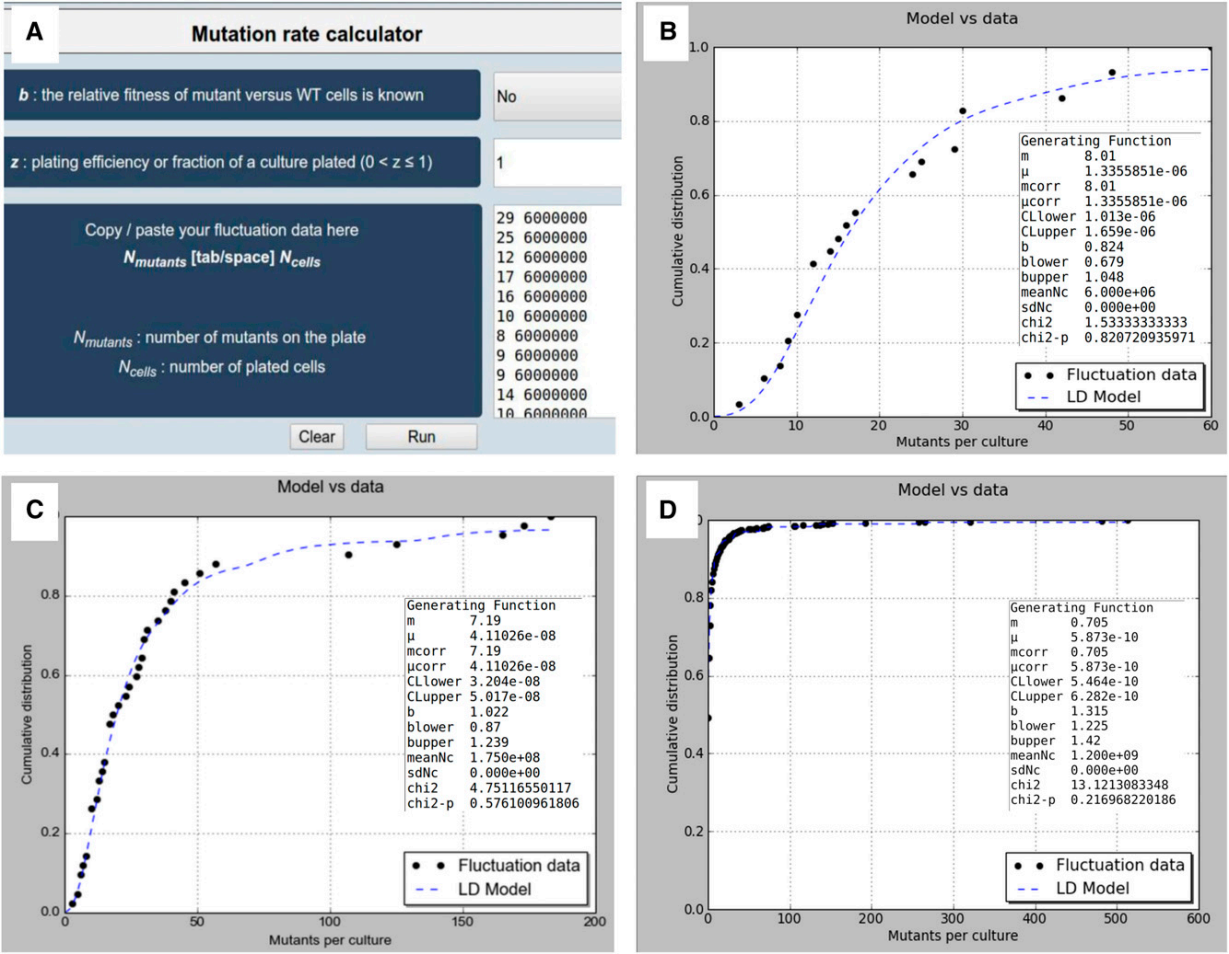
Screen shots of the *bz-rates* Web site. (A) The input form is composed of one choice-field (for the *b* parameter) and two boxes [for the *z* parameter and a two-columned data box (*N_mutants_* and *N_cells_*)]. If the user chooses to manually specify a value for *b*, a supplementary box appears below the choice field. The *z* parameter is the plating efficiency which represents the fraction of a culture plated. The *N_mutants_* and *N_cells_* box is intended to enter the number of plated mutants and plated cells in each culture, respectively. $N_{mutants}$ and *N_cells_* must be spaced by a single white-space or a tabulation. Here, the *N_mutants_* and *N_cells_* box is filled with the values from our experimental fluctuation assay described in the result section. (B−D) Each result section is composed of a numerical box (inside the plot) and a plot showing the cumulative distribution function fitted to the experimental data: (B) results from our experimental fluctuation assay, (C) results from a Luria and Delbrück fluctuation analysis of mutations conferring virus resistance in bacteria [corresponding to the pool of experiments number 1, 10, 11, 15, and 21 from Table 2 in (Luria and Delbrück 1943)], and (D) results from a fluctuation experiment of mutations conferring nalidixic acid resistance in *Escherichia coli* from Boe *et al.* (1994).

*bz-rates* is composed of a simple form (Figure 1A). The first choice field provides the user with the possibility to indicate that *b* is known. In this case, the *b* field appears and the experimentally determined value of *b* can be filled in (0<b<∞). Otherwise, *b* will be estimated computationally by the GF.

The second box allows to fill in the plating efficiency *z* (*i.e.*, the proportion of cells from each culture that was plated, default value: *z* = 1).

The main field is the “*N_mutants_* *N_cells_*” box that parses the fluctuation analysis counts. *N_mutants_* and *N_cells_* are the number of plated mutants and plated cells per culture, respectively. This field is Excel-ready; thus, counts can be directly copy/pasted into this box without further formatting.

The *bz-rates* result section is composed of two parts: the numerical and the graphical boxes (Figure 1). The numerical box on the left provides the following estimates:

- *m*: mean number of mutations per culture not corrected by the plating efficiency (*z*)
- *μ*: mutation rate per cell per division not corrected by the plating efficiency (*z*)
- *m_corr_*: number of mutations per culture corrected by the plating efficiency (*z*)
- *μ_corr_*: mutation rate per cell per division corrected by the plating efficiency (*z*)
- *CL_lower_*: lower 95% confidence limit for *m_corr_*
- *CL_upper_*: upper 95% confidence limit for *m_corr_*
- *b*: mutant cells relative fitness predicted by the GF (only output if *b* is left empty in the input field)
- *b_lower_*: lower 95% confidence limit for *b*
- *b_upper_*: upper 95% confidence limit for *b*
- $\bar{Nc}$: average number of plated cells per culture
- σ_*Nc*_: standard deviation of the number of plated cells
- χ^*2*^: Pearson’s chi-square value
- χ^*2*^ − *pval*: Pearson’s chi-square p-value

The graphical box plots the cumulative distribution function fitted to the experimental data. It allows the user to visually judge for the correctness of the hypothesized distribution. To quantify the quality of the fit, *bz-rates* performs a Pearson’s chi-square goodness-of-fit as described in Boe *et al.* (1994). If the null hypothesis is rejected (p-value<0.01), the user is advised by a red warning that the predicted and observed distributions are not in close agreement. In this case, the user should consider using another model that takes into consideration other deviations from the Luria Delbrück model such as, for instance, the postplating growth (Lang and Murray 2008). To do so, the user should use an advanced mutation rate calculation packages to explore different models such as Salvador (Zheng 2008b).

### Experimental testing

A fluctuation assay was performed with a yeast strain carrying a genetic system that is designed to generate a functional copy of an auxotrophic gene when the cells undergo a specific chromosomal rearrangement (a reciprocal translocation, see *Materials and Methods*). The resulting mutant cells have a strong growth defect relatively to wild-type cells, probably as the result of the translocation, that was experimentally measured to 0.76 (*Materials and Methods*).

The form of Figure 1A is filled with the data of the 30 tubes fluctuation assay that was undertaken. We neglected to specify the relative growth rate of the mutants to compare the predicted relative growth rate of *bz-rates* to the experimental measure. Figure 1B shows *bz-rates* results. The plot indicates a good fit between the statistical distribution of the mutants and the experimental data. Pearson’s chi-square goodness-of-fit value (1.16) and p-value (0.88) are displayed at the end of the numerical box on the left. The mutation rate (μ) is estimated to 1.33 × 10^−6^ per cell per division (95% confidence limits 1.01 × 10^−6^ to 1.6 × 10^−6^ ) and the predicted mutant relative growth rate [0.82 (0.68−1.05)] is in close agreement with the experimental measure (0.76).

### Published datasets and simulations

To test our implementation and the stability of the GF estimator in *bz-rates*, we tested two published datasets: (i) the first dataset corresponds to a historical fluctuation assay composed of 42 parallel cultures performed by Luria and Delbrück (Figure 1C). With this dataset, *bz-rates* predicts a mutation rate (4.11 × 10^−8^) close to the one calculated by Luria and Delbrück (1943) (2.48 × 10^−8^). The value of *m* (7.19) and *b* (1.022) are very close to the range of values reported in Hamon and Ycart (2012) [(5.22−8.89) for *m* and (0.74−1.22) for *b*]. We also tested one larger fluctuation dataset from Boe *et al.* (1994) that is composed of 1102 cultures (Figure 1D). In this case, *bz-rates* reports a *m* value of 0.705, which is close to the one calculated in Hamon and Ycart (2012) (0.65−0.77) and the one calculated in Zheng (2005) (0.71). The mutant differential growth rate value is 1.315, which is a bit greater than the one reported by Zheng (2005) with a ML approach (*b* = 1.193).

**Figure 2:**
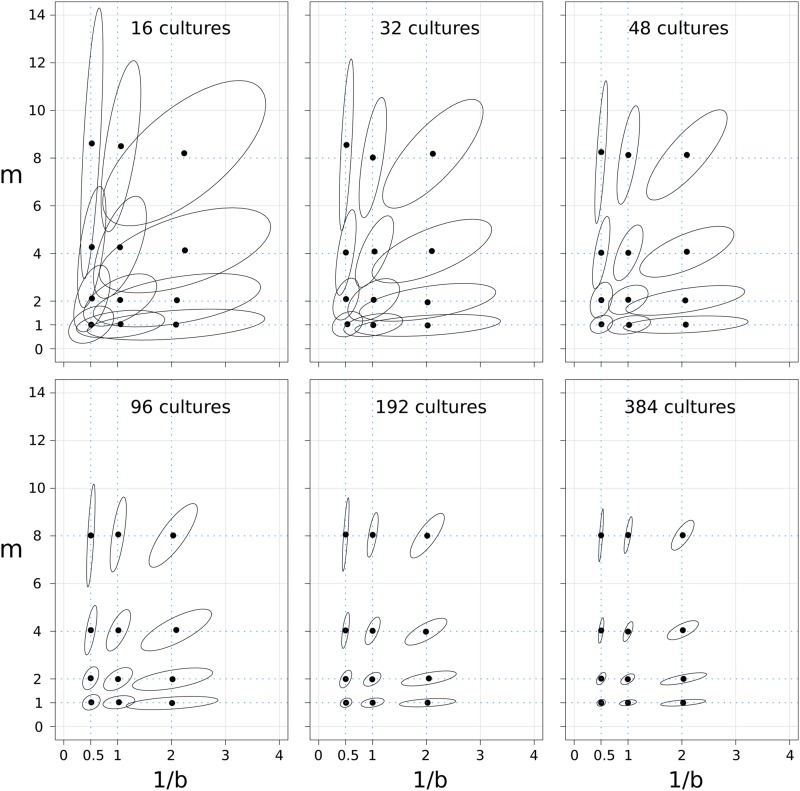
Performance of the *bz-rates* calculator on various simulated datasets. Each panel corresponds to simulated fluctuation datasets with either 16, 32, 48, 96, 192, or 384 independent cultures. In each panel, 200 simulations were performed for different values of *m* (1, 2, 4, and 8) and *b* (0.5, 1, and 2). The ellipses show the 95% dispersion of *bz-rates* estimations for the 200 simulations.

The performance of *bz-rates* also was tested on simulated datasets. We generated simulated fluctuation assays for different couples of *m* and *b* with either 16, 32, 48, 96, 192, or 384 parallel cultures (Figure 2). As expected, the precision of the estimator increases with increasing numbers of parallel cultures. The general trend that can be inferred from these plots is that the precision on the estimation of *m* (and by consequence the estimation of μ) is higher for the smallest values of *m*. Therefore, users should not outgrow the cultures in order to limit the number of mutants that grow on selective plates.

Note that the GF estimator has also been extensively tested elsewhere (Hamon and Ycart 2012; Ycart 2013) and the reader should refer to these papers for an extensive review of the performance of this estimator.

*bz-rates* is a Web tool that does not require the installation of any third party tool or run any command line to estimate mutation rates. It has a minimalist design in order to provide biologists with a web-tool the most straightforward as possible. To our knowledge there was so far a single web-tool available for mutation rate calculation (Hall *et al.* 2009) but this tool does not allow to consider deviations from Luria Delbrück or to estimate the goodness of fit with the model. Therefore, *bz-rates* provides useful advances such as accounting for two important deviations to Luria-Delbrück distributions (*b* and *z*) as well as giving an indication of the reliability of the estimated mutation rates. We hope that *bz-rates* will reveal useful to a broad community of microbiologists and geneticists.
